# The state of telepathology in Japan

**DOI:** 10.4103/2153-3539.68327

**Published:** 2010-08-10

**Authors:** Takashi Sawai, Miwa Uzuki, Akihisa Kamataki, Ikuo Tofukuji

**Affiliations:** 1 Division of Leading Pathophysiology, Department of Pathology, School of Medicine, Iwate Medical University, Japan; 2 Department of Pathology, School of Medicine, Toho University Faculty of Medicine, Japan; 3 Department of Healthcare Informatics, Faculty of Health and Welfare, Takasaki University of Health and Welfare, Japan

**Keywords:** Japan, telepathology

## Abstract

Telepathology began in Japan in the early 1990s in response to advances in computing and telecommunications equipment development and a dearth of pathologists. Telepathology in Japan is most often used for rapid intraoperative pathological diagnosis using frozen section, followed by second opinions and consultation. Intraoperatively, telepathology is used to determine malignancy, metastasis of malignant tumors, and the extent of excision. Infrastructure and equipment has evolved from analog lines to digital lines like integrated services digital network (ISDN) and asymmetric digital subscriber line (ADSL), and recently to fiber optics. The use of communications satellites is also being considered. Image quality is being improved to Hi-Vision (HDTV), and from still images to real-time video. Digital microscopy has been introduced, and is used in education and consultation.

## STATE OF PATHOLOGY IN JAPAN

**Figure 1:**
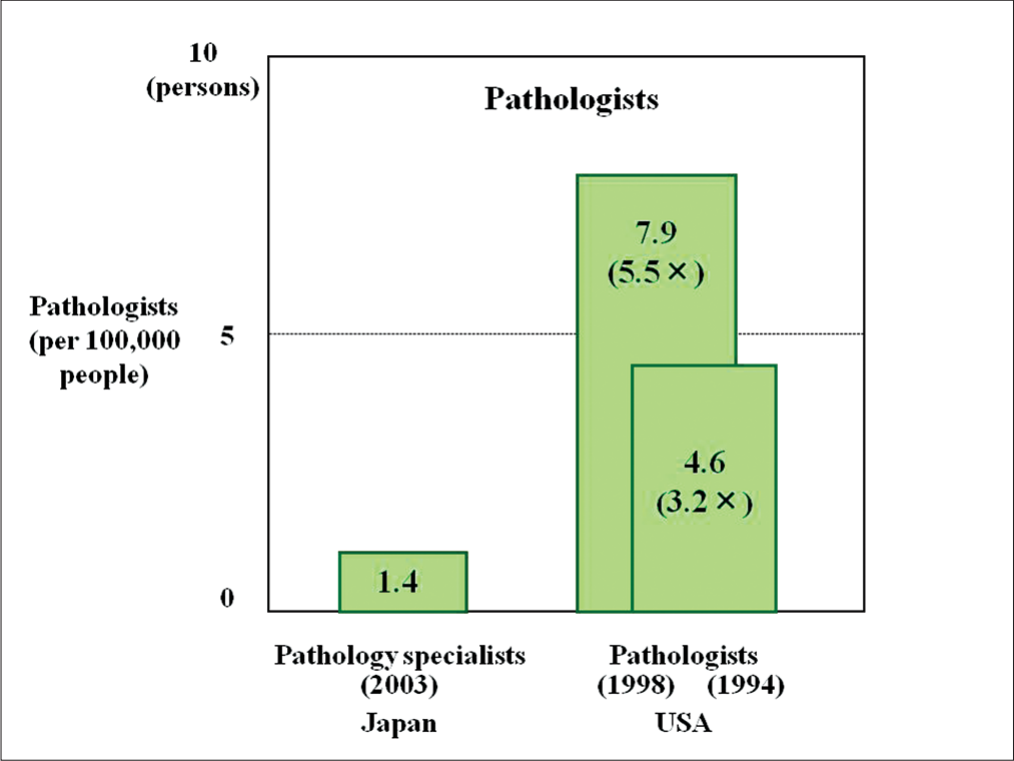
Number of pathologists in Japan and the United States. The number of pathologists in Japan (per 100000 persons) is a mere 1/5 of that in the United States

**Figure 2:**
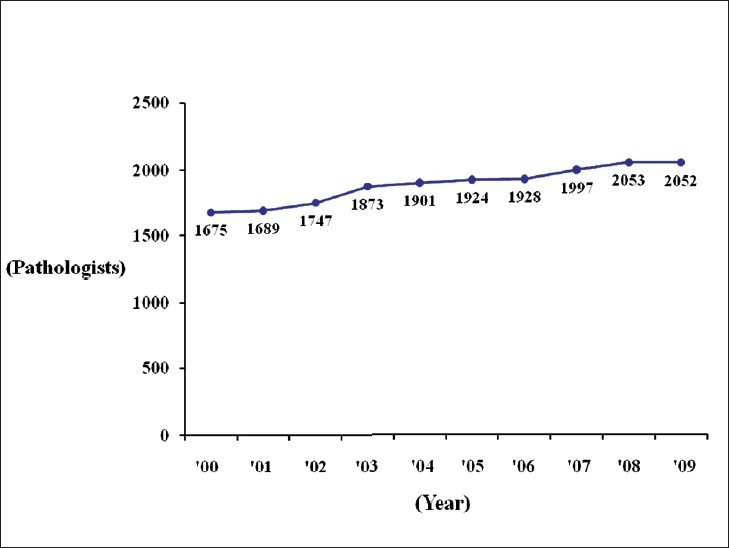
Change over time in the number of pathologists in Japan. Though the number of pathologists is slightly increasing annually, the total is barely 2000

**Figure 3:**
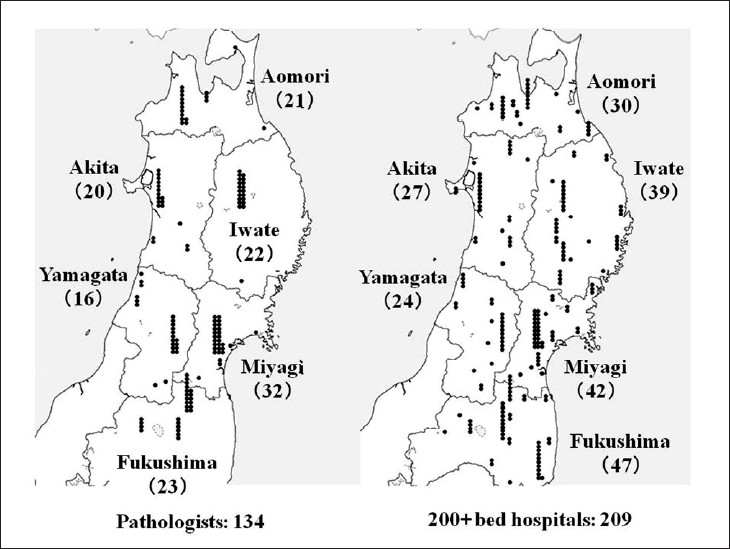
Distribution of pathologists in Tohoku region hospitals. Though the Tohoku region (northeastern Honshu) has 209 hospitals of at least 200 beds, there are only 134 pathologists to serve the area

**Figure 4:**
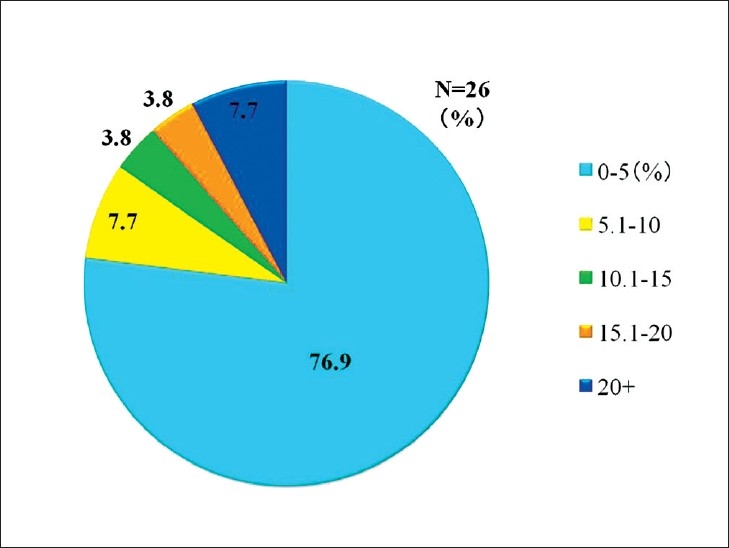
Percentage of operations for which surgeons desire rapid pathological diagnosis. The average is 5%, with some range

**Figure 5:**
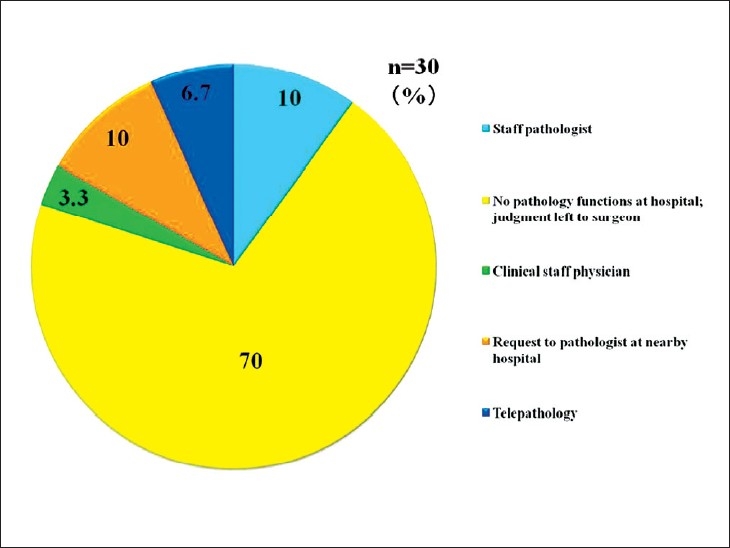
Rapid pathological diagnosis procedures by facility. An overwhelming 70% of cases are handled by surgeons

**Figure 6:**
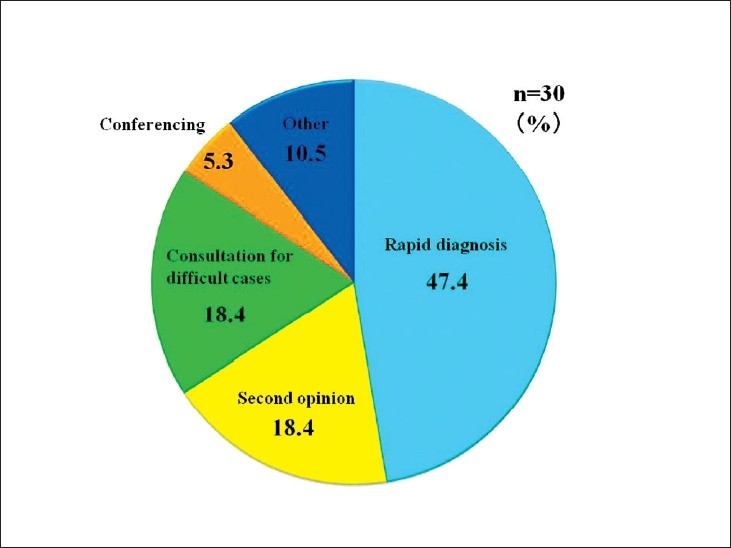
Objectives of telepathology. Eighty three percent of telepathology use is for intraoperative rapid diagnosis, second opinions, and consultations on cases that are difficult to diagnose

Telepathology was developed in Japan using computing and telecommunications equipment to respond to a shortage of diagnostic pathologists. Before addressing telepathology itself, it is important to quickly outline the state of diagnostic pathology in Japan. As of 2010, there are 2052 diagnostic pathologists recognized by the Japanese Society of Pathology (JSP), accounting for only 0.7% of the total number of physicians in Japan and showing only minimal annual growth.[1] This is the severest specialist shortage of any medical field in Japan, followed in order by pediatricians, OB/GYNs, and anesthesiologists. As illustrated in Figure 1, the number of pathologists to the general population is only about 1/5 of that in the United States. Pathologists have traditionally performed autopsies, biopsies, cytodiagnoses, and rapid diagnosis. More recently, they also provide clinicopathological conferences (CPCs) for residents and clinicians. The most recent available JSP study (2006) indicates that Japan’s pathologists perform 25 000 autopsies, 4.7 million biopsies, 8.6 million cytodiagnoses, and 130 000 rapid diagnoses annually. Other than autopsies, all of these numbers are increasing[2] [Figure 2]. Japan has more small-scale hospitals than most other countries. The situation in the Tohoku region (northeastern Honshu) is illustrative. Despite having 209 hospitals with 200 or more beds, full-time pathologists are almost exclusively confined to university hospitals and major hospitals in the prefectural capitals [Figure 3]. Even larger hospitals in other major cities rarely have full-time staff pathologists.[1] Medical facilities without full-time pathologists often outsource biopsies and cytodiagnoses to university, public or private laboratories. Results are generally available in ten days or so, making intraoperative rapid diagnosis using frozen section impossible. Thus, decisions on the extent of excision are left to surgeons’ experience and intuition. A veteran surgeon’s judgment can be quite accurate, but new and unknown cases or borderline lesions can make even experienced doctors hesitate. In the case of cancer, tumors, not fully excised will recur. A survey of surgeons with 15 or more years of experience revealed that in 3–10% of past cases (5% on average), these doctors wished they had been able to perform intraoperative rapid diagnosis[1] [Figure 4]. In hospitals without pathologists, about 70% of surgeons decide the operative course depending on clinical experience rather than histopathological findings [Figure 5]. Given this situation, the government began promoting intraoperative rapid diagnosis using computing and telecommunications equipment as a way to improve the level of medical treatment in Japan. Though globally telepathology is most frequently used for consultation and gathering second opinions, in Japan use of and expectations for intraoperative rapid diagnosis are highest[3]. [Figure 6].

## HISTORICAL OVERVIEW OF THE DEVELOPMENT OF TELEPATHOLOGY IN JAPAN

In 1982, Japan’s first telepathology experiment linked Keio University to Ise Keio Hospital (both in Tokyo) via an analog phone line.[3] Almost a decade later at the 23rd Japan Medical Congress in 1991, the Kyoto Prefectural University of Medicine connected with Yosanoumi Hospital (on the Japan Sea side) to demonstrate telepathology. Intraoperative telepathological rapid diagnosis was subsequently added to the university’s normal operations. Additionally, the National Cancer Center hooked up its main hospital with Hospital East (both in Tokyo) via fiber-optic cable, and Yamagata University connected its faculty of Medicine and University Hospital with fiber optics as well. At the 81st meeting of the JSP (1992), Tohoku University was fiber optically linked with Sendai City Hospital for a video telepathology demonstration.[4] However, in the early 1990s each facility was using unique telepathology formats.[5]

**Figure 7:**
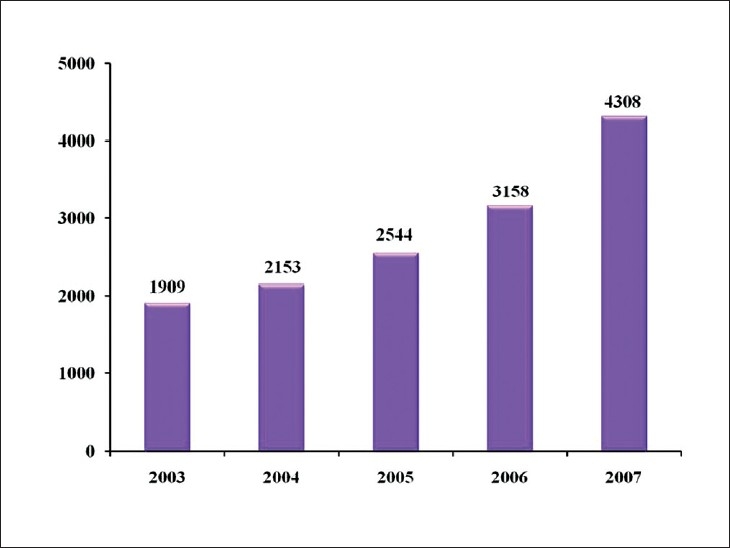
Cases of telepathology use. Telepathology use is increasing annually in Japan, topping 4000 cases in 2007

The dissemination of telepathology has accelerated recently due to societal factors such as the continuing shortage of diagnostic pathologists, advances in information technologies (IT) and digital devices, and the spread of the Internet on the one hand, and patients’ increasing awareness of malpractice and desire for a second opinion on the other. In addition, the work of the telemedicine research group established in 1996 by the Ministry of Health, Labor and Welfare (MHLW) cannot be ignored. From the outset, this research group researched fields including tele-homecare, teleradiology, and telepathology. Subsequent significant events in the history of telepathology include telepathology’s addition as an insured healthcare service in 2000, and the expansion of telepathology facilities in 2003. Of particular importance is that the MHLW’s official acceptance of telepathology represented a change from its previous policy of recognizing only direct and face-to-face medicine. This was a major impetus for the spread of telepathology. Though additional fees for remote services (such as added equipment and telecommunication fees) are still not officially recognized, recent surveys have shown that the usage of telepathology is gradually increasing. At present, at least 40 or so facilities are linked with about 120 hospitals and clinics to provide telepathological services for nearly 4300 cases annually[6] [Figure 7].

## OBJECTIVES OF TELEPATHOLOGY USAGE IN JAPAN

**Figure 8:**
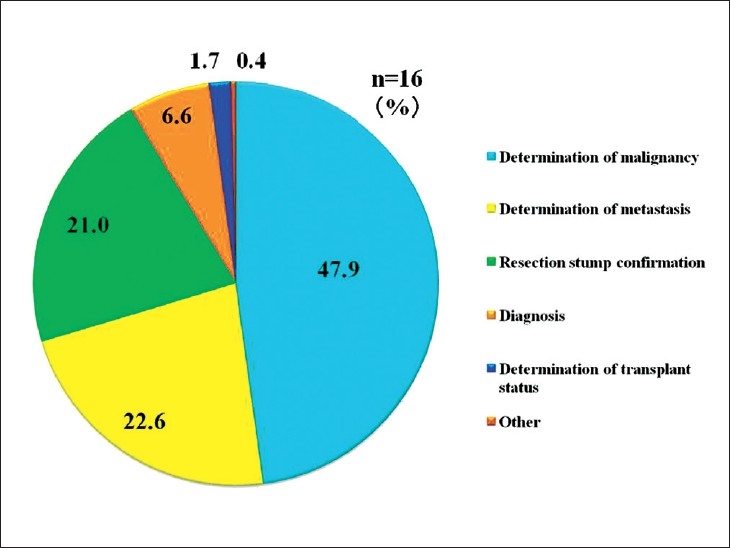
Rapid diagnosis breakdown. Telepathology is used to determine malignancy, metastasis of malignant tumors, and the extent of excision

**Figure 9:**
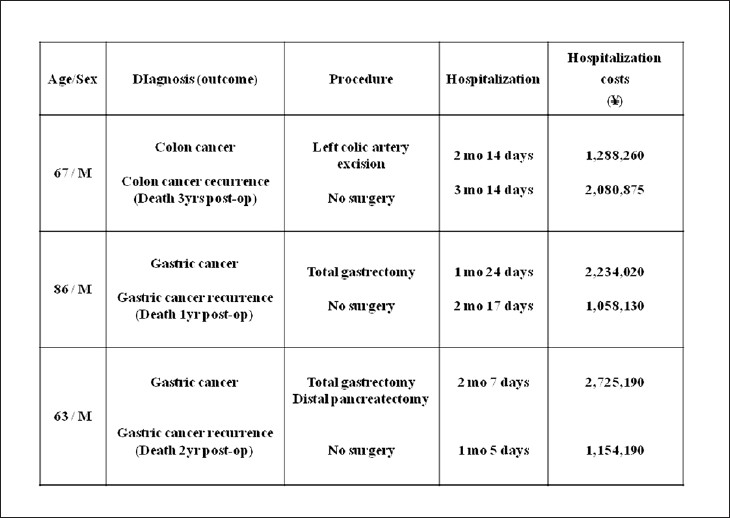
Examples of financial burden in cases of cancer recurrence. The financial burden for recurrence is often nearly equal to or even greater than for initial operations. Additionally, human resources are tied up and patient deaths not uncommon

In Europe and the United States, telepathology is used widely in consultations.[7] However, in Japan it is overwhelmingly used for intraoperative rapid diagnosis.[3] There are two reasons for this difference. The first is that telepathology in Japan began from rapid diagnosis under the auspices of the MHLW, designed to increase parity in medical care. The other is the latent desire for rapid intraoperative pathological diagnosis that telepathology fulfills for clinicians, especially surgeons. Rapid telepathological diagnosis is used to diagnose malignant tumor and metastasis, and to confirm resection margin[8] [Figure 8]. Improperly or incompletely removed tumors always recur, endangering patient’s lives. But that is not all. Recurrence obviously places enormous physical, emotional, and financial burden on patients and their families, and also wastes valuable medical time and resources. Studies have shown that initial operations on gastrointestinal cancers (such as stomach and colon cancer) cost about 2 million yen, and that subsequent operations for recurrence tend to be equally expensive[1] [Figure 9]. On the other hand, using video-assisted thoracoscopic surgery (VATS) for rapid lung cancer diagnosis and moving directly to excision as part of the same operation in the case of malignancy leads to savings of 500,000 yen over performing two separate surgeries (VATS to make a paraffin section for diagnosis, followed several days later by tumor excision when diagnostic results become available).[9] It is clear that the addition of intraoperative rapid diagnosis not only improves patients’ prognosis, but has a significant positive economic effect.

In addition to intraoperative rapid diagnosis, the uses of telepathology in Japan include second opinions, consultation, and conferencing. Second opinions are sought mostly for judgments on the malignancy of tumor, lesions and the course of surgery. Consultations are generally made regarding borderline lesions (those for which cancer diagnosis is difficult) rather than for cases that are more difficult to diagnose. In other words, telepathology consultations are used to determine whether surgery is required immediately or whether additional time can be taken before the decision to operate. In case of breast cancer, many doctors expressed a desire for second opinions for both diagnosis and determining the course of surgery.[10] Clinicopathological conferences (CPCs) are not yet practiced at most facilities, but with the introduction of the medical-internship system in Japan and the overall shortage of diagnostic pathologists in Japan, conferences should be seriously considered. For these reasons, telepathology will continue to be indispensable to cope with Japan’s shortage of diagnostic pathologists while maintaining parity and high quality in medical care.

## INFRASTRUCTURE AND TELEPATHOLOGY SYSTEMS IN JAPAN

Telepathology systems require both hardware and software. In the case of telepathology, hardware is mainly IT-dependent and includes digital cameras, computers, and microscopes, while software refers primarily to computer applications.

**Figure 10:**
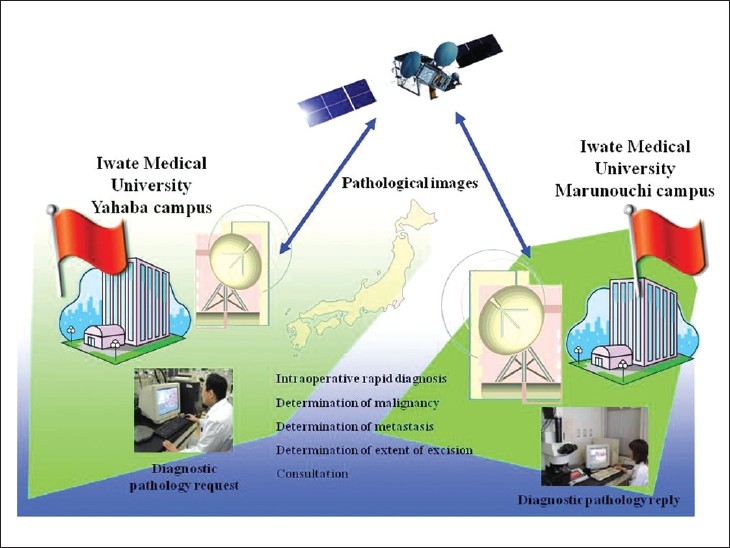
Overview of telepathology experiment via communications satellite. In January 2010, successful telepathology experiments were carried out using video and digital slides over high-speed communications satellites

In its infancy, telepathology relied entirely on analog phone lines. When ISDN subsequently became available, single and then multiple, bundled ISDN lines were used. Subsequently ADSL became standard, but recently the field has progressed to fiber-optic cable infrastructure, vastly increasing data transfer rates and volume. Mobile telepathology using communications satellites is also in development, allowing mobile phones and other devices to receive image data on the move. We are currently experimenting with video and virtual slides using an ultra-high-speed Internet communications satellite (Wideband InterNetworking engineering test and Demonstration Satellite “KIZUNA” (WINDS)); however, issues including image quality, operability, and internationalization remain unresolved[11] [Figure 10].

**Figure 11:**
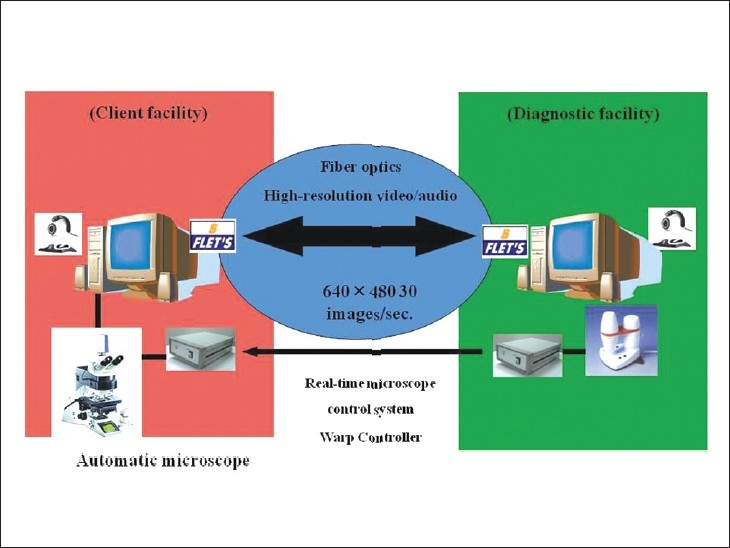
Real-time fiber-optic telepathology. Diagnosticians (pathologists) can freely operate a real-time fiber-optic telepathology system like this, selecting the field of view, adjusting the focal point, etc.

Currently, the overwhelming majority of telepathology systems in Japan rely on the transfer of still images over ISDN lines. Though in most cases, the provider (pathologist) is able to select the field of view for observation, some systems still require the client (physician requesting diagnosis) to operate the system. Analog telepathology formats using telephone lines still exist, but they are disappearing rapidly. In their place, formats using high-speed broadband Internet connections (ADSL and fiber optics) have appeared and are gaining popularity [Figure 11]. In particular, fiber-optic video telepathology allows diagnosticians to select the viewing field and operate the equipment, making the observation process nearly identical to checking specimens under a conventional microscope.[12,13] In recent years, as part of its efforts to ensure equal treatment for cancer patients, the government has promoted the use of digital microscopy in both diagnostic pathology and consultation, providing subsidies that have helped digital microscopy spread across Japan.[14] However, digital microscopes, while widely used in education, are not currently used with great frequency in rapid intraoperative pathological diagnosis because they require more loading time than video. Digital microscopes are used at approximately 250 facilities nationwide, including 50 universities (about 60% of universities with medical departments). At half of these facilities, digital microscopes are used in histology and histopathology training. They are also used by non-university hospitals for histological specimen preservation and conference presentations.

## FUTURE ISSUES FOR TELEPATHOLOGY IN JAPAN

**Figure 12:**
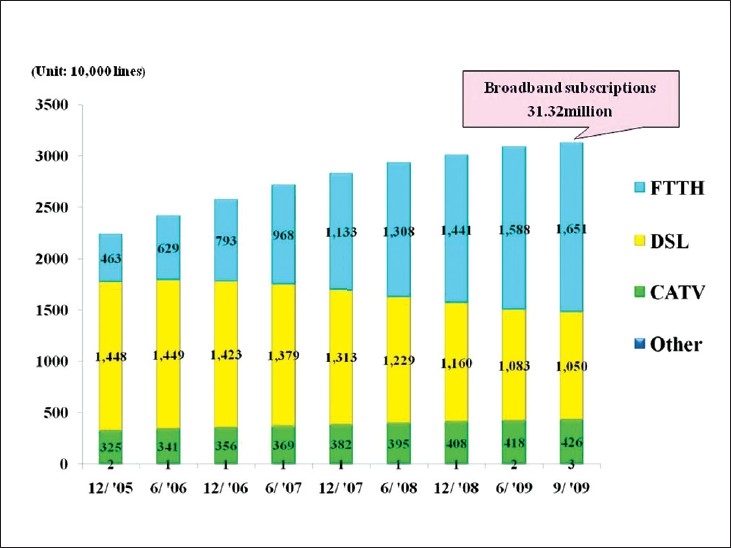
The spread of broadband access in Japan. There were 30 million broadband subscribers in Japan in 2009

**Figure 13:**
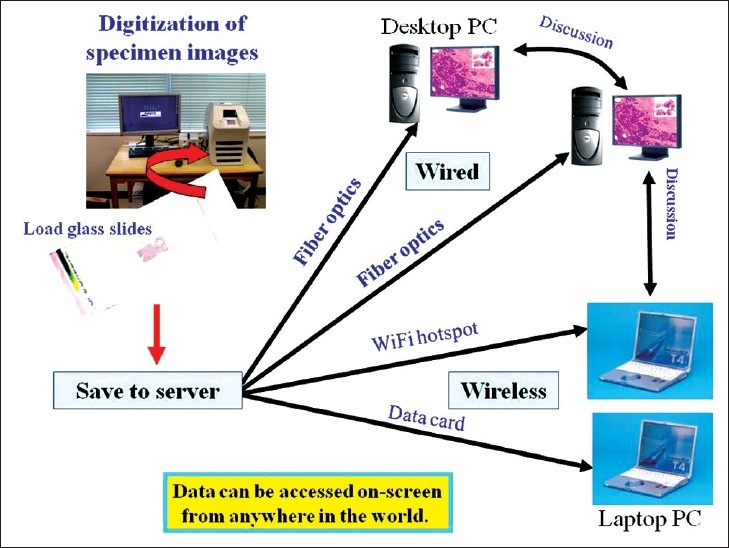
Consultation using digital microscopy. Consultations use digital microscopy images saved to a server. With proper authentication (ID and password), data can be accessed from anywhere in the world

The spread of telepathology in Japan has been promoted by government policies and advances in IT [Figure 12]. We are currently in the age of fiber optics, which has allowed us to predicate development of telepathology on high-speed broadband connections. Internationally, the infrastructure differences remain, but telepathology is expected to continue to grow.[15] It seems likely that telepathology using digital microscopy may well become the norm [Figure 13].

**Figure 14:**
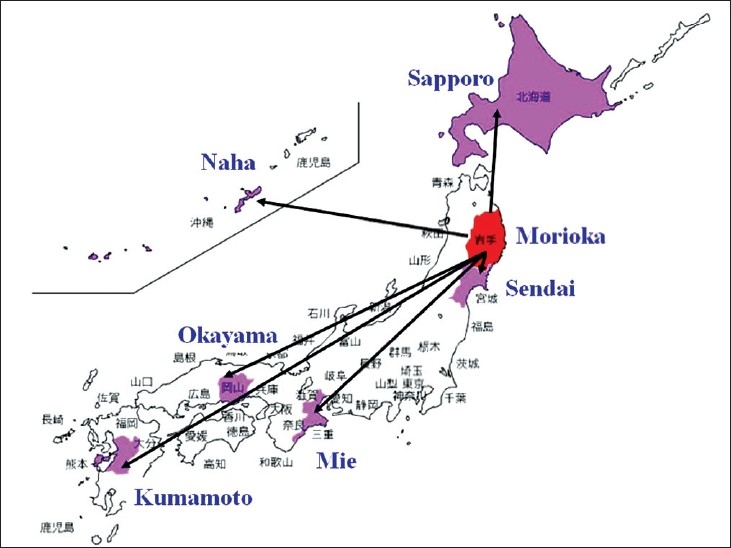
Example of request for consultation using digital microscopy. This example of a digital microscopy consultation request in Japan illustrates a simultaneous request to multiple facilities

**Figure 15:**
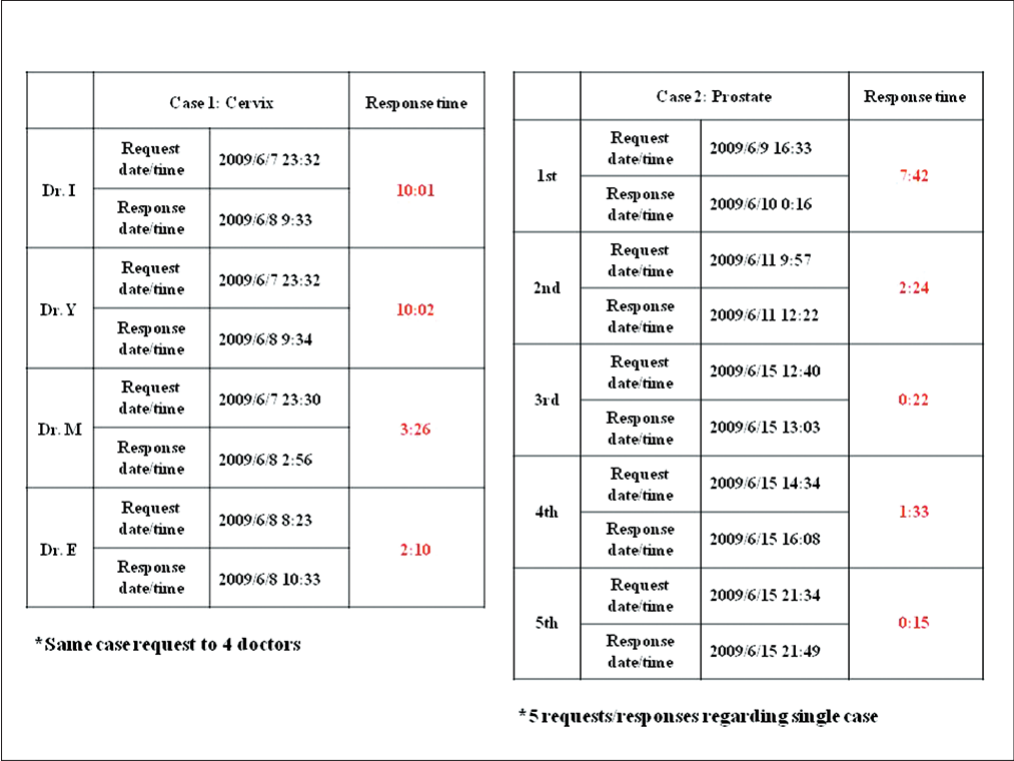
Actual consultation response times. Responses to consultation requests were received in 2–10h. A reply was received in 10min in the case of an identical case sent using a digital slide

In Japan, education, workshops, consultations, and cytodiagnosis using digital microscopy has already begun.[16,17] At present, fiber optics are not available in all areas, meaning that Internet connections over normal digital lines are used in many cases; however, this is slowly changing. When broadband Internet becomes ubiquitous, ideally, we will be able to toggle between real-time video and uploaded images with a single-click so that both can be used like digital microscopy in rapid diagnosis, teaching, and consultations. Recently, we constructed a fiber optic digital microscopy consultation system. With this system, we can simultaneously consult on difficult cases with multiple pathologists regardless of distance or national borders [Figure 14], and can quickly receive responses from multiple consultant pathologists [Figure 15]. This system is highly economical and labor efficient.

Telepathology in Japan began as an expedient way to use IT to compensate for the shortage of diagnostic pathologists. In this sense, the progress of telepathology is a major scientific achievement and an important part of Japan’s national IT strategy. Should the number of diagnostic pathologists increase, manpower issues could be overcome. However, such an increase is highly unlikely anytime soon. The continuing advancement of medicine, medical lawsuits stemming from the problematic diagnoses, national policies promoting a move to electronic medical records, and the increasing use of images in e-learning all mean that image-based telepathology is poised for continued growth and development with the improvement of related infrastructure and hardware.
